# Circadian Rhythms of Body Temperature and Locomotor Activity in Spontaneously Hypertensive Rats under Frequent Changes in Light Conditions

**DOI:** 10.3390/pathophysiology31010010

**Published:** 2024-03-01

**Authors:** Anna Yu. Ryabinina, Anna A. Bryk, Mikhail L. Blagonravov, Vyacheslav A. Goryachev, Andrey A. Mozhaev, Vera S. Ovechkina

**Affiliations:** 1 V.A. Frolov Department of General Pathology and Pathological Physiology, Institute of Medicine, Peoples’ Friendship University of Russia (RUDN University), 6 Miklukho-Maklaya Street, 117198 Moscow, Russiablagonravov-ml@rudn.ru (M.L.B.);; 2 Shemyakin-Ovchinnikov Institute of Bioorganic Chemistry, Russian Academy of Sciences, Miklukho-Maklaya, 16/10, 117997 Moscow, Russia; a.a.mozhaev@gmail.com (A.A.M.);

**Keywords:** core body temperature, locomotor activity, spontaneously hypertensive rats, telemetry, chronobiology, cosinor, circadian rhythm

## Abstract

Changes in lighting accompany modern urbanization trends and can lead to various pathologies based on circadian disturbances. In this study, we assessed the changes in the circadian rhythm of core body temperature (Tcore) and locomotor activity of Wistar-Kyoto rats (WKY) and spontaneously hypertensive rats (SHR) following exposure to different lighting conditions: extended light phase of the day (16 h–8 h, 20 h–4 h, 24 h–0 h), light pollution, monochromatic light, and bright light therapy. The telemetry data was collected after experimental lighting conditions during periods with standard lighting (12 h of light and 12 h of darkness) and was processed using linear and cosinor analysis. The daily rhythms of rats’ parameters persisted in accordance with the standard lighting regime. Tcore changes were observed in both groups compared to the initial period: in WKY, a decrease in Tcore during the darkness and an increase during the light; in SHR, the opposite trend, with Tcore increased during the darkness and decreased during the light phase of the day. A relationship between Tcore and activity was observed with weak correlation. WKY exhibited more pronounced signs of adaptive variation and desynchronization compared to SHR, which could be associated with a wider range of functional capabilities of the organism without cardiovascular pathology.

## 1. Introduction

The modern pace of life is often characterized by abrupt changes in light exposure. These changes are driven by numerous factors such as the use of artificial lighting, crossing time zones during flights, night shift work schedules, and others. Circadian rhythms, representing endogenous variations in biological processes over a 24 h period, are closely linked to the light–dark cycle of the day. Environmental surroundings, social and urbanization factors, irregular sleep patterns, and somatic pathologies can contribute to circadian system disruptions [1]. Circadian disturbances are associated with various illnesses and reduced quality of life, necessitating the application of various therapeutic methods, including chronomedicine techniques such as light therapy [2].

The circadian timing system comprises central clocks in the suprachiasmatic nucleus and peripheral clocks in various tissues [1]. These clocks interact through neural, endocrine, temperature, and behavioral signals, and each of these systems autonomously responds to central clocks [1,2]. Furthermore, alterations in the light–dark cycle can resynchronize circadian gene expression in the pineal gland over a period of 7 days [3]. Studies of desynchronization indicate that disruptions in circadian rhythms may occur due to the disconnection of neuronal oscillators in the suprachiasmatic nucleus [4].

Circadian rhythms are characteristic of most physiological systems in the body, including thermoregulation [5,6], which has a complex regulatory mechanism [7,8]. In rats leading an active nocturnal lifestyle, an increase in body temperature is typical during the dark phase of the day, while a decrease occurs during the light phase [9]. Modulation of body temperature rhythm is primarily regulated by the circadian oscillator of the suprachiasmatic nucleus, as evidenced by the persistence of circadian rhythms in forcibly desynchronized rats [4]. It is known that, in rodents, core body temperature and locomotor activity are well synchronized [10].

Alterations in body temperature and locomotor activity rhythms in rats are observed during surgical interventions [11], hypoxic exposure [12], dietary changes [13,14], physical exercise [15,16,17], and various forms of stress [18].

Additionally, the influence of lighting on the body temperature cycle has been noted [19]. Rats exhibit disruptions in circadian rhythms of temperature, locomotor activity, and the activity of the pineal N-acetyltransferase and estrous cycles when the standard 12–12 h light–dark cycle is shifted to continuous lighting [20]. Moreover, the circadian rhythmicity of melatonin levels in the bloodstream of rats was maintained during prolonged continuous lighting, while the circadian rhythms of temperature and locomotor activity became arrhythmic [21]. In rats constantly exposed to light, the connection between circadian melatonin secretion and biological clocks becomes more stable than the connection between locomotor activity and body temperature [22]. Prolonged daytime illumination (20:00–24:00) over several weeks results in decreased animal temperature and activity [23]. Nighttime light pollution, a modern urbanization phenomenon, contributes significantly to disrupting biorhythms [24]. It is worth noting that, in addition to the lighting regimen, the wavelength of light also influences changes in circadian rhythms, as demonstrated in studies involving monochromatic light [25,26].

Thermoregulation is linked to processes involving changes in vascular tone, and temperature fluctuations often serve as predictors of decompensation in hypertensive conditions [27]. Mechanisms of cardiovascular control play a central role in the integrated regulation of both arterial pressure and body temperature [28].

Rats with spontaneous hypertension (SHR—Spontaneously Hypertensive Rat) demonstrate autonomic imbalance and abnormal body temperature regulation [29]. It should be noted that the increase in blood pressure in animals of the SHR line begins at the age of 6 weeks, reaching systolic pressure between 180 and 200 mm Hg in adulthood [30]. The role of changes in lighting in the thermoregulation of SHR remains unknown. Therefore, the primary aim of our study is to elucidate how alterations in lighting affect thermoregulation in SHR. The results of our research may contribute to advancing knowledge about the molecular and mechanistic aspects of circadian pathways, their role in pathophysiological processes, and the development of new chronotherapeutic methods, representing a multifaceted medical challenge.

## 2. Materials and Methods

### 2.1. Animals and Environmental Conditions

The experiment involved 10 male rats, divided into two groups: 5 spontaneously hypertensive rats (SHR) and 5 Wistar-Kyoto rats (WKY) serving as the control group. The animals were obtained from the “Pushchino” Laboratory Animal Nursery, a branch of the Shemyakin-Ovchinnikov Institute of Bioorganic Chemistry of the Russian Academy of Sciences. At the onset of the experimental study, the ages of the animals were 34–36 weeks.

For 14 days prior to the commencement of the experiment, including the surgical intervention for sensor implantation, the animals were kept in conditions ensuring a light–dark cycle of 12 h of light and 12 h of darkness, conforming to the standard housing conditions for this type of laboratory animals. During the experiment, each rat was housed in an individual cage measuring 15 cm in height, 21 cm in width, and 38 cm in length. Artificial lighting was maintained in the facility, ensuring free movement and unrestricted access to water and food for the animals. Feeding was carried out daily at the same time—19:00. Animals received the regular adapted rat food: laboratory feed for rodents (full-fledged extruded feed for keeping laboratory animals).

The temperature in the facility where the animals were housed was maintained at a constant +22 ± 2 °C, which is optimal for maintaining the normal physiological conditions of these animals.

The experiment was carried out in accordance with the European Convention for the Protection of Vertebrate Animals used for Experimental and Other Scientific Purposes (Strasbourg, 18 March 1986) and was also approved by the Ethical Committee of the RUDN Institute of Medicine (Approval Code: #3).

### 2.2. Study Design

During the entire course of the experiment, the animals were subjected to various light regimes. The experimental regime lasted for 3 days, followed by 3 days under a standard light regime. The standard artificial light–dark pattern with a ratio of light and dark phases 12–12 h (light phase—from 07:00 h. to 19:00 h., lighting 350 lux at the level of the animals’ eyes, provided by a luminescent light source with a color temperature of 4500 Kelvin; darkness—from 19:00 h. to 07:00 h., lower than 0.5 lux). The standard 12–12 h lighting regime in the study was selected to investigate potential delayed effects of experimental lighting on the temperature and activity of rats during standard lighting conditions. We aimed to assess whether physiological parameters recover to baseline values or undergo other changes, understand the dynamics of these changes, explore the adaptability of animals to rapid shifts in lighting conditions, and evaluate the impact of short periods of light therapy. The use of a standard 12–12 lighting cycle for three days was specifically chosen to examine the completeness of adaptation and its effectiveness in preventing desynchronosis in animals under these conditions.

After the surgical procedure of biotelemetry sensor implantation, the animals had an adaptation period of one week under standard light conditions. After their recovery, recording of the indicated parameters began, and for the first 3 days, the animals were kept under a standard light regime, which we will call Period 1.

Subsequently, the light phase of the day was extended: for the next 3 days, lights were on from 5:00 h. to 21:00 h., constituting a regime of 16 h of light and 8 h of darkness. The following week simulated a regime of 20 h of light and 4 h of darkness (light phase from 3:00 h. to 23:00 h., dark phase from 23:00 h. to 3:00 h.). After these light regimes, the animals were once again kept under the standard light regime, referred to as Period 2.

For the next 3 days, the animals were exposed to monochromatic light with a wavelength of 520 nm during the light phase, generated by a full-spectrum luminescent light source incorporating a light filter that selectively transmitted the dominant wave-length at 520 nm. This was followed by Period 3 of the standard light regime.

The next experimental light regime involved light pollution. In this regime, animals were kept under the standard light regime of 12 h of light and 12 h of darkness, but during the dark phase, the illuminance at the eye level of the animal was maintained at 1.5 lux. This was again followed by the standard light regime, referred to as Period 4.

Subsequently, the animals were subjected to a bright light therapy regime for 3 days, which involved exposure to cold light with an intensity of 9000 lux at the animals’ eye level for 1 h (from 10:00 h. to 11:00 h.) using full-spectrum LED lamps with a color temperature of 6500 Kelvin. This was followed by the standard light regime, referred to as Period 5.

Finally, the last experimental regime involved continuous illumination (24:0 h) for 3 days, after which the animals were returned to the standard light regime (Period 6).

**Figure 1:**
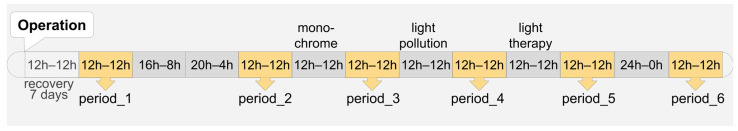
Periods of continuous telemetry data analysis (light–darkness hours in one day). Postoperative recovery period lasted 7 days, after which telemetry data was collected by periods. Each period lasted 3 days, in which the same light regime was observed; for example, 16 h of light–8 h of darkness during the day (“16 h–8 h” block in the diagram). The light regimes of all periods changed continuously. Telemetry data were assessed on the middle day of each period. A total of six periods were analyzed.

Data were assessed and compared only during the periods with the 12 h light and 12 h darkness regime (Periods 1–6) (Figure 1).

### 2.3. Core Body Temperature and Activity Telemetry

Continuous registration of core body temperature (Tcore) and activity was carried out with biotelemetric monitoring technique using the radio-telemetry system (Data Sciences International, St. Paul, MN, USA). For this purpose, DSi HD-S11 radio transmitters were implanted into the animals surgically under general anesthesia (Zoletil, Virbac, Carros, France). A midline laparotomy of about 3 cm was performed, and a transmitter was placed into the abdominal cavity and fixed to the anterior abdominal wall during its suturing. The wound was closed in layers. Monitoring of Tcore and activity started 7 days after surgery. Animals were kept in individual cages with free access to water and food.

Radio transmitters are devices that monitor BP, biopotentials of the heart, Tcore, and activity and transmit data as a radio-signal to special receivers placed near the animals’ cages.

The thermal sensor is located in the housing of the radio transmitter, which, after completion of the operation, remained in the abdominal cavity of the animal. Mean Tcore is calculated based on averaging the Tcore over 20 sub-segments of 60 s segment length. The default reduced mean calculation discards outliers in the sub-segments in the calculation. The frequency of recording mean Tcore was every 60 s with an accuracy of 10^−10^ °C.

Activity measurement—any signal indicating relative animal activity movement. Source could be animal movement detected by a receiver, lick sensor activity, etc. The signal strength is used to derive activity counts. An algorithm is applied to derive these activity counts based on the amplitude and speed of the change in signal strength. The activity counts are a relative measure of activity and cannot be quantified to a distance moved or speed of movement. The telemetry software (Dataquest ART 4.2 Gold Acquisition Software—Data Exchange Matrix, Data Sciences International, St. Paul, MN, USA) processes the signal strength using an algorithm to calculate activity counts based on thresholds of signal strength changes over time. Activity sources are reported in units of counts per minute. The activity counts are derived over each scheduled sampling interval (60 s) for scheduled sampling. Activity values are always scaled to counts per minute regardless of the averaging interval (Dataquest A.R.T. 4.2 User Guide—Theory of Operation, Data Sciences International, St. Paul, MN, USA).

Recording of the mentioned parameters was started 7 days after the implantation of transmitters.

### 2.4. Data Analysis

#### 2.4.1. Linear Data Analysis

Telemetry data of 24 h (from 07:00 h to 07:00 h of the next day) in the middle of each period was used to carry out linear analysis including calculation of mean, standard error of mean (SEM), coefficient of variation (CV), and Spearman correlation. To discern group and period differences, mean values and utilized bootstrapping for confidence intervals, alongside boxplots for visual interpretation, were examined. All parameters were also calculated separately for light (from 07:00 h to 19:00 h) and dark (from 19:00 h to 07:00 h) day phases. The degree of decrease in Tcore and activity in rats during daylight hours was calculated using the daily index (DI).

DI = (Mean of data(darkness) − Mean of data (light))/Mean of data (darkness) × 100%

To assess the extent of variation in parameter values, the relative variation indicator was used, which is measured in relative terms compared to the average level. CV is expressed as a percentage ratio of the standard deviation to the mean. The CV value can be used to evaluate the degree of data homogeneity. The greater its value, the greater the spread of characteristic values around the mean, the less homogeneous data in composition, and the less representative the mean.

#### 2.4.2. Circadian Rhythm Assessment

The circadian rhythm was assessed using telemetry data from all three days of each period, assessed by linear analysis.

Nonlinear regression analysis of telemetry data was carried out using cosinor-based rhythmometry [31]. The cosinor parametric method based on trigonometric regression (cosine approximation) estimates the rhythm parameters, which describes the circadian rhythms with an algorithmic pattern or sinusoidal waveform. A rhythm is a regular periodic component in a time sequence, summarized through parameters: mesor, amplitude, and acrophase [32].

In the current study, the circadian rhythm of Tcore and activity was also assessed using these parameters of multicomponent cosinor analysis of a 24 h period:

- Mesor (midline estimating statistic of rhythm)—the average level of the data in the 24 h period, calculated as a sum of minimum value and a half of a difference between maximum and minimum parameter value in the 24 h period;
- Amplitude—maximum deviation of the corresponding indicator from the mesor, calculated as a half of a difference between maximum and minimum parameter value in the 24 h period;
- Acrophase—a measure of the time of the general high values of the function repeated in each cycle, expressed in (negative) degrees with respect to a reference time set at 0°, with a period corresponding to 360° (2π or 6.283185 radian); in multicomponent analysis, it is defined as the first peak of the cycle;
- Ortophase (from lat. orto—rise)—phase of the cycle with the maximum value of the function (maximum peak), used in multicomponent analysis; may be the same value as acrophase.

The number of cosinor functions (components) approximating telemetry data in multicomponent analysis was determined by automatic selection of the best model regarding the number of components performed using the extra sum-of-squares F-test [33]. Changes in the circadian rhythm were determined using these cosinor parameters, including phase fluctuations also known as “phase shift”.

#### 2.4.3. Statistics

The significance of the differences in the means was checked using the Mann–Whitney U test (the difference in the mean values was taken as significant at *p* ≤ 0.05).

#### 2.4.4. Software

Biotelemetry recordings were acquired every minute and analyzed using Dataquest ART 4.2 Gold Acquisition and Analysis Software (Data Sciences International, St. Paul, MN, USA).

Most of the modern available software requires additional manual processing of primary research data and does not meet the stated goals in many research parameters. Taking into account large amounts of telemetry data and specific processing for rhythm analysis, an application for this study was created and approved at the V.A. Frolov Department of General Pathology and Pathological Physiology, Peoples’ Friendship University of Russia. Data analysis was carried out using the software based on the Python 3.10 packages: CosinorPy for cosinor-based rhythmometry [33,34] and Pandas, SciPy, NumPy, Mathplotlib, etc., for statistical and linear data analysis and plotting.

## 3. Results

### 3.1. Tcore Telemetry

#### 3.1.1. General Analysis of Tcore Data

The core body temperature (Tcore) significantly differed between light and dark phases of each day in all periods in both groups of rats (*p*-value < 0.01), with a noted lower level of Tcore during the light phase of the day.

**Figure 2:**
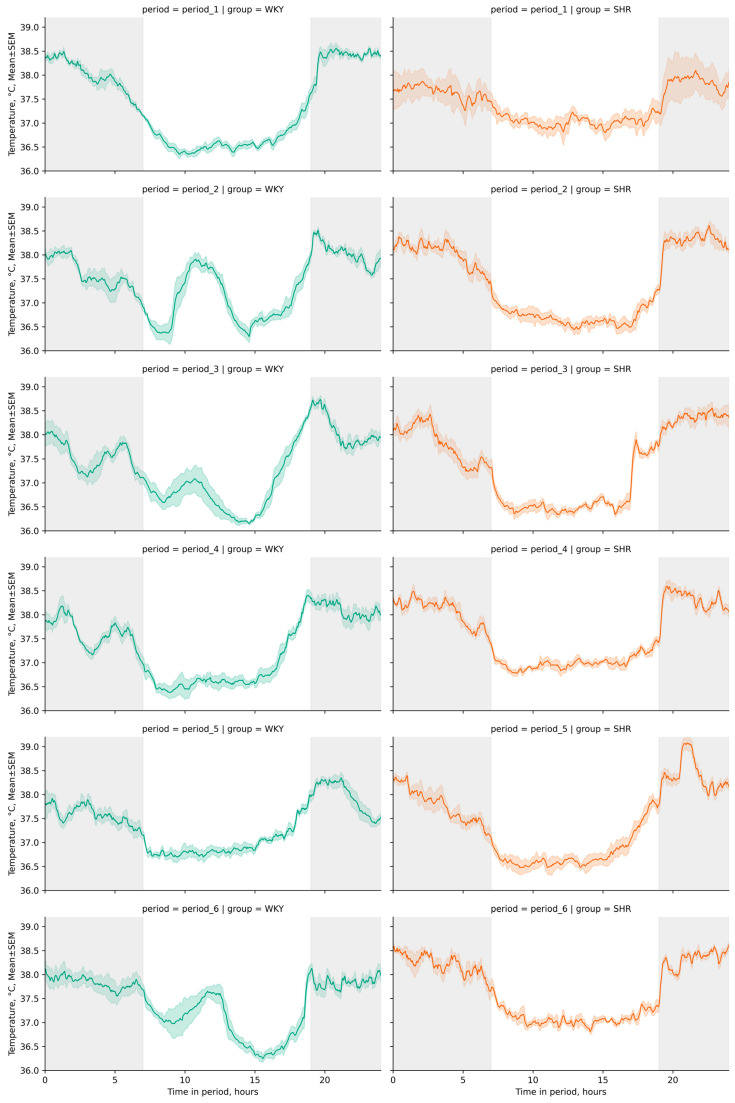
Line plots of Tcore fluctuations across 24 h cycles by periods (period_1–6) and groups (WKY and SHR). Throughout the telemetry data analysis, a standard artificial light–dark cycle was maintained in each period, with dark phases spanning from 19:00 h to 07:00 h (12 h) represented by a gray background and light phases from 07:00 h to 19:00 h (12 h) denoted by a white background. The evaluation periods under standard lighting conditions were subsequent to days exposed to various experimental lighting conditions: period_1 followed a postoperative recovery week, period_2 followed days following a 20 h light and 4 h darkness regime, period_3 followed a monochromatic light regimen, period_4 followed days affected by light pollution, period_5 followed a regimen of bright light therapy, and period_6 followed continuous illumination (24:0 h). Notably, lower levels of Tcore were recorded during daylight hours in both groups. In WKY, discernible peaks in Tcore were observed during the light phases of the second, third, and sixth periods, indicating a sharp increase in Tcore.

The daily index (degree of Tcore reduction during the light phase of the day) was 3.89 ± 0.19, 1.99 ± 0.34, 2.37 ± 0.24, 2.62 ± 0.18, 2.04 ± 0.18, and 2.26 ± 0.17% for WKY and 1.78 ± 0.6, 3.71 ± 0.15, 3.47 ± 0.13, 3.03 ± 0.12, 3.25 ± 0.26, and 3.01 ± 0.2% for SHR in each respective period. These distinctive features in the daily fluctuations of Tcore, typical for most rodents leading an active nocturnal lifestyle, were visually discernible on linear graphs of rat Tcore during the studied periods (Figure 2).

**Figure 3:**
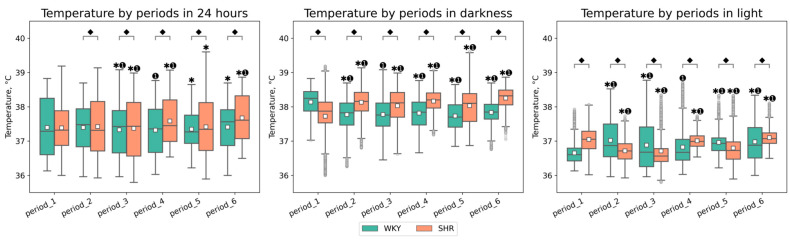
Tcore values in 24 h, darkness, and light by periods and groups of rats. The evaluation periods under standard lighting conditions were subsequent to days exposed to various experimental lighting conditions: period_1 followed a postoperative recovery week, period_2 followed days following a 20 h light and 4 h darkness regime, period_3 followed a monochromatic light regimen, period_4 followed days affected by light pollution, period_5 followed a regimen of bright light therapy, and period_6 followed continuous illumination (24:0 h). ⬥ *p* < 0.05 WKY vs. SHR in one period, ✱ *p* < 0.05 vs. previous period, ➊ *p* < 0.05 vs. first period; □—Mean value.

Following experimental lighting conditions, certain tendencies in Tcore changes were observed in both groups compared to the initial baseline period: in WKY, we observed a decrease in Tcore during the dark phase and an increase during the light phase, whereas in SHR, we observed the opposite trend, with Tcore increased during the dark phase and decreased during the light phase of the day (Figure 3). Additionally, peaks in Tcore during the daytime were observed in WKY—A sharp increase in Tcore during the light phases of the second, third, and sixth periods (Figure 2). The most significant fluctuations in daytime Tcore were observed in both groups after lighting regimes that involved an increase in the light phase and a decrease in the dark phase (16–8, 20–4, 24–0 h regimes), as well as after monochromatic lighting. SHR exhibited higher Tcore during the dark phase compared to WKY in all studied periods except the first one. However, during the light phase, such distinct tendencies were not observed—the differences in Tcore between WKY and SHR had varying characteristics in each time period (Table 1, Figure 3).

#### 3.1.2. Variation of Tcore Data

**Figure 4:**
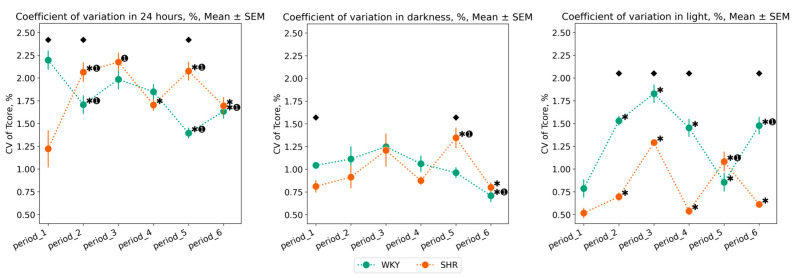
Tcore coefficient of variation (CV) in 24 h, dark, and light phase of the day by periods and groups of rats. The evaluation periods under standard lighting conditions were subsequent to days exposed to various experimental lighting conditions: period_1 followed a postoperative recovery week, period_2 followed days following a 20 h light and 4 h darkness regime, period_3 followed a monochromatic light regimen, period_4 followed days affected by light pollution, period_5 followed a regimen of bright light therapy, and period_6 followed continuous illumination (24:0 h). Data are expressed as means ± SEM; ⬥ *p* < 0.05 WKY vs. SHR in one period, ✱ *p* < 0.05 vs. previous period, ➊ *p* < 0.05 vs. first period.

The daily variations in Tcore appear after conducting experimental lighting—from the second to the fifth period, in SHR, there is an increase in CV, whereas in WKY, there is an overall decrease over 24 h. Compared to WKY, SHR exhibits a lower degree of Tcore variation, especially during the daytime: CV of Tcore during the light phase was higher in WKY in most periods after experimental lighting regimes (periods 2, 3, 4, 6). However, in the fifth period (following the light therapy regime), there was a sharp decrease in Tcore variation in WKY, practically returning to the initial values, i.e., those of the first period. In contrast, during the fifth period, there was an increase in CV for both the light and dark phases in SHR. After the 24–0 h light–dark regime, the sixth period showed an opposite pattern: a reduction in Tcore variation in SHR and an increase in WKY during the light phase. There were practically no significant fluctuations in the CV of Tcore during the nighttime, except for an increase in CV during the fifth period in SHR and a decrease in CV during the last period in WKY (Figure 4).

#### 3.1.3. Cosinor Analysis of Tcore Data

**Figure 5:**
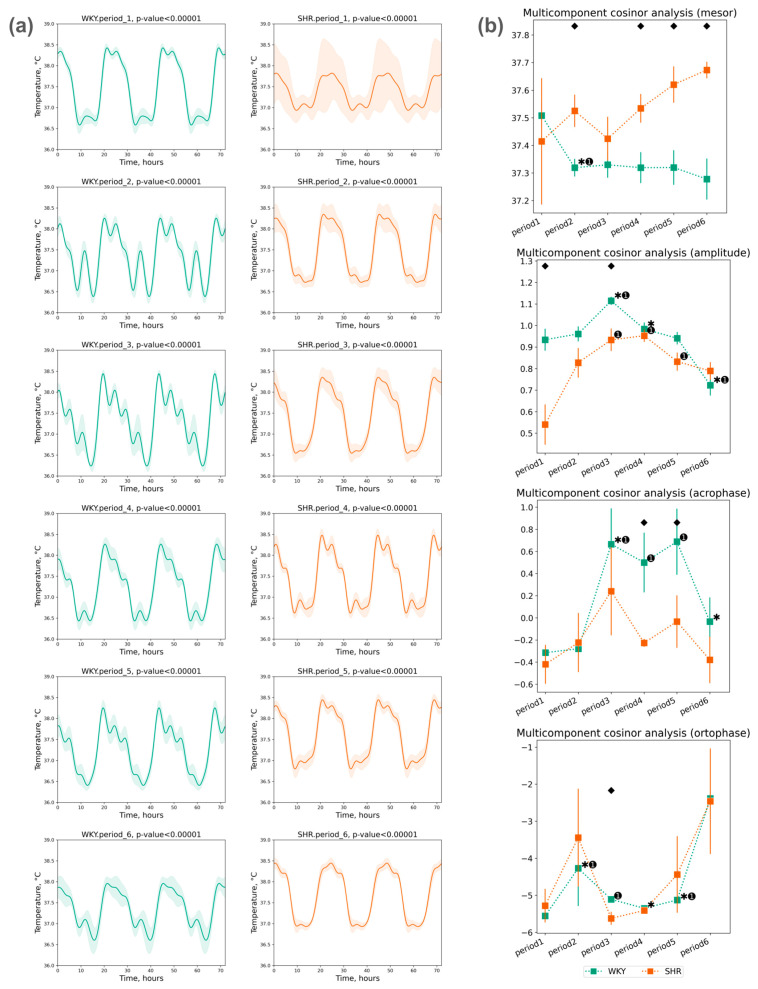
Cosinor analysis of Tcore: (**a**) Tcore circadian rhythm of 3 days in periods approximated by multicomponent cosinor (component = 5); (**b**) Measurements of circadian rhythm multicomponent cosinor analysis of Tcore data in 3 days by periods and groups (component = 5): mesor (in °C), amplitude (in °C), acrophase (in radians: phase of ±2π) and ortophase (in radians: phase of ±2π). The evaluation periods under standard lighting conditions were subsequent to days exposed to various experimental lighting conditions: period_1 followed a postoperative recovery week, period_2 followed days following a 20 h light and 4 h darkness regime, period_3 followed a monochromatic light regimen, period_4 followed days affected by light pollution, period_5 followed a regimen of bright light therapy, and period_6 followed continuous illumination (24:0 h). Data are expressed as means ± SEM; ⬥ *p* < 0.05 WKY vs. SHR in one period, ✱ *p* < 0.05 vs. previous period, ➊ *p* < 0.05 vs. first period.

The assessment of Tcore rhythm was conducted using a five-component cosinor analysis over three days within each selected period. A significant circadian rhythm was reliably detected in each period (Figure 5a).

The mesor values of Tcore did not exhibit significant deviations throughout the periods. There was a tendency towards an increase in the Tcore mesor in SHR and a significant decrease in WKY during the second period, with this level being maintained in all subsequent periods. The amplitude of Tcore did not significantly differ in the second period; however, subsequent changes were noted: an increase in amplitude in the third period (after monochromatic lighting), followed by a trend of decreased amplitude in both groups in the fourth and subsequent periods. The following phase changes were identified:

- The acrophase shift of Tcore was noted during the third, fourth, and fifth periods compared to the initial (first) period in WKY, while no significant acrophase shift was observed in SHR;
- The ortophase shift of Tcore was also noted only in WKY during the second to fifth periods (Figure 5b).

### 3.2. Activity Telemetry

#### 3.2.1. General Analysis of Activity Data

**Figure 6:**
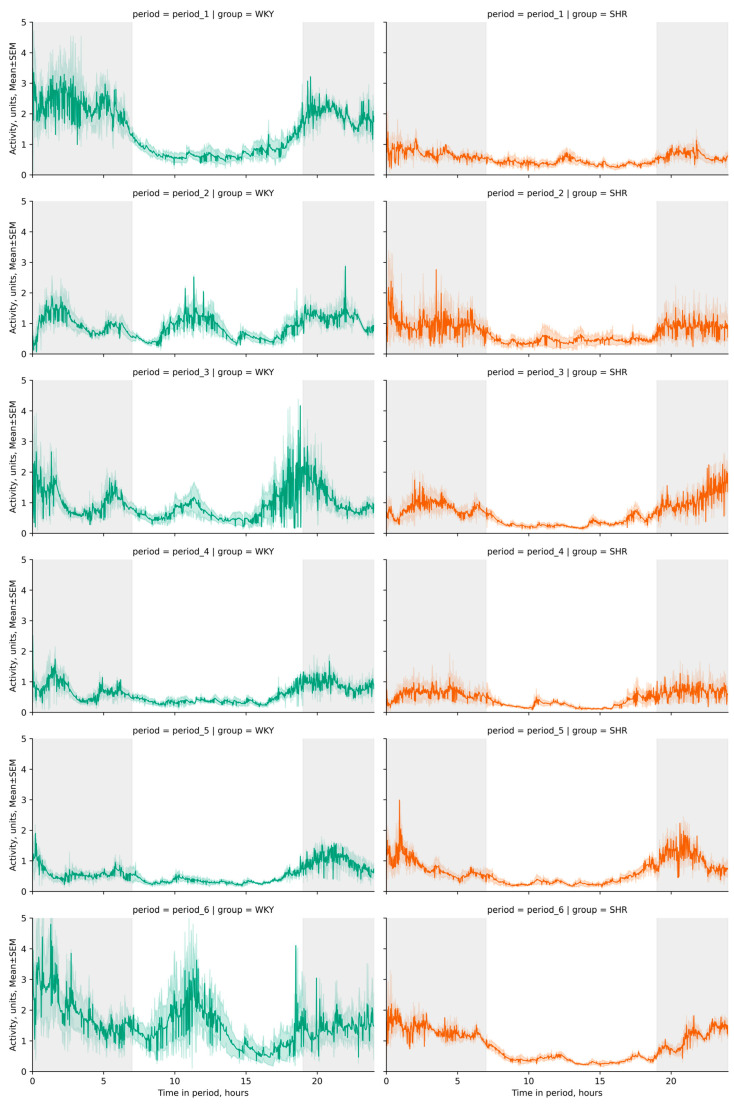
Trend line plots of activity fluctuations across 24 h cycles by periods (period_1–6) and groups (WKY and SHR). Throughout the telemetry data analysis, a standard artificial light–dark cycle was maintained in each period, with dark phases spanning from 19:00 h to 07:00 h (12 h) represented by a gray background, and light phases from 07:00 h to 19:00 h (12 h) denoted by a white background. The evaluation periods under standard lighting conditions were subsequent to days exposed to various experimental lighting conditions: period_1 followed a postoperative recovery week, period_2 followed days following a 20 h light and 4 h darkness regime, period_3 followed a monochromatic light regimen, period_4 followed days affected by light pollution, period_5 followed a regimen of bright light therapy, and period_6 followed continuous illumination (24:0 h). Notably, lower levels of activity were recorded during daylight hours in both groups. In WKY, discernible peaks in activity were observed during the light phases of the second, third, and sixth periods, indicating a sharp increase in activity. Activity data out of IQR was filtered out with the following exponentially weighted calculations (span = 100) for plotting due to high variation and extremely noisy waveforms of original data.

**Figure 7:**
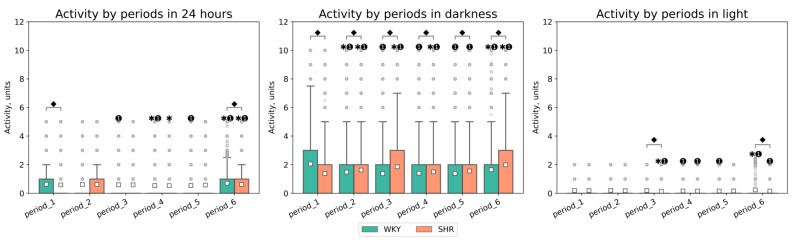
Activity values in 24 h, darkness, and light by periods and groups of rats. The evaluation periods under standard lighting conditions were subsequent to days exposed to various experimental lighting conditions: period_1 followed a postoperative recovery week, period_2 followed days following a 20 h light and 4 h darkness regime, period_3 followed a monochromatic light regimen, period_4 followed days affected by light pollution, period_5 followed a regimen of bright light therapy, and period_6 followed continuous illumination (24:0 h). ⬥ *p* < 0.05 WKY vs. SHR in one period, ✱ *p* < 0.05 vs. previous period, ➊ *p* < 0.05 vs. first period; □—Mean value. Activity data out of IQR was filtered out.

The activity significantly differed between light and dark phases in both groups (*p*-value < 0.001), with decreased activity observed in both groups during the light phase. The daily index was 60.1 ± 4.2, 15.59 ± 18.77, 13.25 ± 12.12, 38.05 ± 6.0, 39.67 ± 9.41, 22.1 ± 11.72% for WKY and 37.16 ± 5.79, 45.73 ± 5.7, 64.57 ± 3,84, 47.22 ± 6,56, 50.79 ± 2.92, 68.82 ± 2.94% for SHR in each respective period. These specific daily fluctuations in rat activity were visually evident on linear graphs of rat activity during the studied periods (Figure 6 and Figure 7). WKY showed a decrease in locomotor activity following experimental lighting conditions compared to the first period.

Peaks in daytime activity were observed in WKY—an increase in activity during the light phases of the second, third, and sixth periods (Figure 6). Significant differences in activity between rat groups were observed during nighttime in all periods, and during daytime in the third and sixth periods. The average activity of WKY during the dark phase was only higher in the first period compared to SHR, whose average activity most significantly differed in the third period (after monochromatic lighting) and the sixth period (after continuous lighting) (Table 2, Figure 7). Critical differences in activity were not observed during other periods.

#### 3.2.2. Variation of Activity Data

Values of CV showed a low degree of data homogeneity after outlier filtering. This likely reflects the absence of significant internal regulation of rat activity—the animal movements were predominantly spontaneous. Despite this, there were several noteworthy aspects in the variation of animal activity.

**Figure 8:**
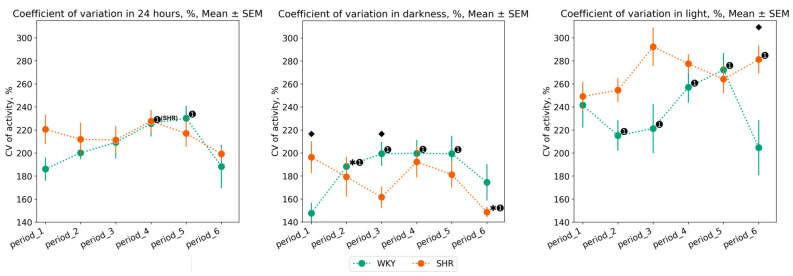
Activity coefficient of variation in 24 h, dark, and light phase of the day by periods and groups of rats. The evaluation periods under standard lighting conditions were subsequent to days exposed to various experimental lighting conditions: period_1 followed a postoperative recovery week, period_2 followed days following a 20 h light and 4 h darkness regime, period_3 followed a monochromatic light regimen, period_4 followed days affected by light pollution, period_5 followed a regimen of bright light therapy, and period_6 followed continuous illumination (24:0 h). Data are expressed as means ± SEM.; ⬥ *p* < 0.05 WKY vs. SHR in one period, ✱ *p* < 0.05 vs. previous period, ➊ *p* < 0.05 vs. first period. Activity data out of IQR was filtered out.

Compared to SHR, WKY exhibited a greater degree of activity variation during the dark phase: WKY’s activity CV during light phases was higher in most periods after experimental lighting regimes (periods 2, 3, 4, 5) compared to the baseline. SHR showed a significant decrease in CV in the last period (after continuous lighting). During the light phase of the day, WKY’s variation decreased in periods 2 and 3, while it increased in periods 4 and 5. Significant changes in activity variation in SHR were observed during the light phase in the last period (Figure 8).

#### 3.2.3. Cosinor Analysis of Activity Data

**Figure 9:**
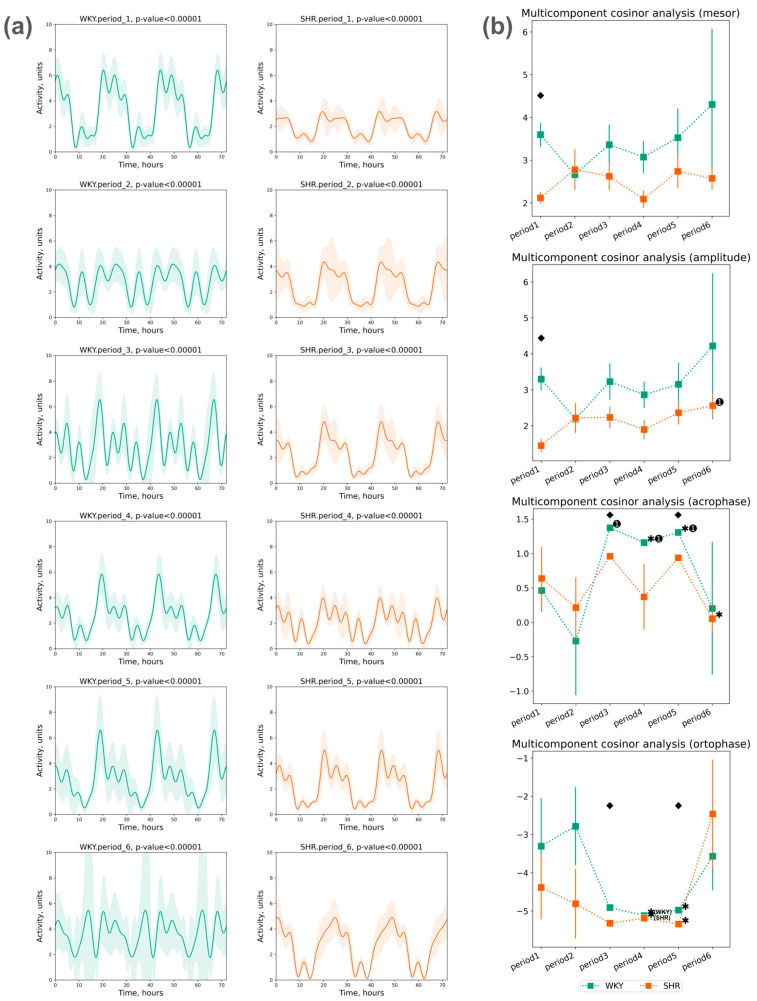
Cosinor analysis of activity: (**a**) activity circadian rhythm of 3 days in periods approximated by multicomponent cosinor (component = 5); (**b**) measurements of circadian rhythm multicomponent cosinor analysis of activity data in 3 days by periods (component = 5): mesor (in °C), amplitude (in °C), acrophase (in radians: phase of ±2π), and ortophase (in radians: phase of ±2π). The evaluation periods under standard lighting conditions were subsequent to days exposed to various experimental lighting conditions: period_1 followed a postoperative recovery week, period_2 followed days following a 20 h light and 4 h darkness regime, period_3 followed a monochromatic light regimen, period_4 followed days affected by light pollution, period_5 followed a regimen of bright light therapy, and period_6 followed continuous illumination (24:0 h). Data are expressed as means ± SEM; ⬥ *p* < 0.05 WKY vs. SHR in one period, ✱ *p* < 0.05 vs. previous period, ➊ *p* < 0.05 vs. first period.

The assessment of locomotor activity rhythm was conducted using a five-component cosinor analysis over three days within each selected period. A significant circadian rhythm of activity was reliably detected in each period (Figure 9a).

The mesor values of activity did not show significant deviations throughout all the periods. The activity amplitude also did not significantly differ across all periods in both groups. The following phase changes were identified:

- The acrophase shift of activity was noted during the third, fourth, and fifth periods compared to the initial (first) period in WKY, while no significant acrophase shift was observed in SHR, except for a deviation in the last period compared to the previous one;
- The ortophase shift of activity was noted in WKY and SHR during the fourth and fifth periods compared to previous periods (Figure 9b).

### 3.3. Correlation of Tcore and Activity Data

A relationship between Tcore and activity was observed in both groups of rats during both light and dark phases of the day. The obtained correlation coefficients from all periods indicate a weak positive linear correlation, except for SHR during daylight in the first and second periods and during the dark phase in the first period, where the correlation test was not significant (Table 3). This could be associated with limited space for rat mobility despite standard housing conditions, leading to noisy data in activity recording, or the presence of non-linear relationships between these physiological parameters, necessitating further investigation.

## 4. Discussion

In this study, we assessed the changes in the circadian rhythm of Tcore (core body temperature) and locomotor activity following exposure to different lighting conditions. Rats exhibited circadian fluctuations in these physiological parameters in all periods in accordance with the standard 12–12 h light–dark cycle and the previously described characteristics of rodents’ active nocturnal lifestyle [35,36]. This suggests a stable endogenous rhythm of Tcore and its accompanying activity rhythm in both rat groups, evidenced by weak variations in the mesor of Tcore across the transition of periods.

However, WKY rats displayed a higher degree of Tcore variation compared to SHR. This might be associated with a broader range of thermoregulatory function fluctuations in WKY, supported by higher amplitude values and a phase shift in the circadian rhythm of Tcore, as well as a peak in Tcore and activity during the light phase of the day in periods following increased light duration and exposure to monochromatic light (periods 2, 3, and 6).

Vascular tone disturbances in SHR likely limit the functional range of thermoregulation fluctuations when exposed to changes in lighting conditions. Moreover, increased amid concurrent peripheral artery vasoconstriction likely contributes to higher internal temperature in SHR during the dark phase compared to WKY, as noted in several studies [8,37]. Another study also suggests a neurogenic resetting of the central thermoregulatory setpoint, resulting in SHR functioning at higher body temperature [38].

Previous research has shown that changes in body temperature are linked to alterations in vascular tone during physical exercise in rats [14]. In our study, Tcore fluctuations show a weak correlation with the activity of rats, despite apparent coincidences in the fluctuation of these parameters over 24 h (Figure 2 and Figure 6). This contrasts with the findings from a study exploring the effects of high-intensity exercise on biological rhythm and demonstrating no influence of physical exertion on the central suprachiasmatic nucleus clock, and a strong correlation between spontaneous physical activity and temperature over a 48 h period [15], which was not observed in our research. This divergence might be due to reduced locomotor capabilities of the rats within the confined space of their cages, despite standard housing conditions. Furthermore, the high degree of variation in locomotor activity mostly indicates its spontaneity and lack of endogenous regulation, as their rhythms are dominated by external factors and the sleep–wake cycle [39]. It is noted that the circadian rhythm of Tcore can be dissociated from rhythmic locomotor activity, partly because the behavioral aspect of rat activity is more strongly influenced by the lighting regime than Tcore itself [4]. Thus, changes in physical activity play an important but not fundamental role in the circadian rhythms of Tcore.

The frequent changes in lighting regimes can be assessed as a stress factor for rats by examining the alterations in temperature and locomotor activity across different phases of the day. Previous studies have shown that, following an acute physical stressor, locomotor activity significantly decreases during the nocturnal (active) cycle, while Tcore markedly increases during the diurnal (inactive) cycle compared to pre-stress levels [16]. In our study, rat activity during the dark phase notably decreased in all periods for WKY during lighting regime transitions, whereas SHR even exhibited increased activity after monochromatic and round-the-clock illumination, and Tcore during the daytime consistently rose alongside its variation in WKY across all periods, whereas SHR predominantly experienced temperature decreases. Consequently, WKY exhibited a more typical stress response to frequent changes in lighting regimes compared to SHR, which might be associated with the weak adaptation mechanisms in SHR. Further investigation, including alterations in hemodynamic parameters, is required, because artificial light regimens not only disrupt the body temperature cycle but also affect the cardiovascular system. It has been demonstrated that constant light induces elevated blood pressure through sympathetic hyperactivity [40]. Based on this data, conclusions can be drawn regarding the disruption of stress responses in SHR, posing a higher risk for distress development and complications associated with arterial hypertension.

Epidemiological studies show increased morbidity and mortality rates in individuals with arterial hypertension [41]. Disruptions in circadian rhythms are associated with a higher risk of cardiovascular diseases, ischemic heart disease, and mortality [42,43]. It has been shown that decreased body temperature might serve as a predictor of cardiovascular complications [28], while alterations in body temperature rhythm amplitude are inversely related to longevity: reduced amplitude correlates with increased mortality [44], whereas increased amplitude is linked to longer life expectancy [45]. In this context, Tcore fluctuations might serve as a biomarker for developing cardiovascular complications.

Experimental increases in light phases throughout the day lead to significant changes in Tcore variation in WKY during daylight hours. Conversely, bright light therapy tends to restore Tcore variation in WKY, almost to baseline values. Currently, bright light therapy is successfully used to treat various pathologies including seasonal and non-seasonal depressions, mood and eating disorders, sleep disturbances, and Parkinson’s disease, among others [46,47,48]. The observed Tcore changes following bright light therapy might be further utilized to enhance the understanding and more effective utilization of this therapeutic method in disease management.

The acrophase shift and changes in cycle amplitude are key factors indicating the presence of desynchronization [31]. Desynchronization refers to a state characterized by a misalignment of previously synchronized intra- or intersystemic rhythms [49,50]. The degree of circadian rhythm desynchronization, characterized by the acrophase and ortophase shifts of Tcore and activity, was most pronounced in WKY following a lighting regime predominantly using the blue part of the light spectrum, light pollution, and light therapy. This suggests an adaptive nature of this desynchronization, rather than a pathological one. In SHR, there were very few significant changes in the parameters of the circadian rhythm of Tcore and locomotor activity, indicating a limited range of physiological variations typical of a pathological hypertensive system.

Previously noted is the slower adaptability of the Tcore rhythm to a new light cycle, which is twice as slow as locomotor activity adaptability [51]. It is essential to emphasize that, within each observed period with alternating 12 h light–dark cycles, lasting for 3 days after experimental lighting regimes, there was no restoration of the initial rhythm parameters. However, no severe disturbances in the circadian rhythm were noted, such as significant mesor shift or inversion of the rhythm phase. This may indicate the stability of endogenous thermoregulatory rhythms in both groups. Considering the influence of hypertension on changes in Tcore rhythm parameters, with the possibility of using them to assess the risks of decompensation of hemodynamic disorders in SHR, represents a new area for further investigation.

However, this study has some limitations. The small number of animals led to a relatively small sample size, impacting the depth of our findings. These factors have been disclosed in the relevant section of our work to ensure transparency and provide a comprehensive understanding of the study’s scope. One notable limitation pertains to the necessity of incorporating additional hemodynamic parameters, such as blood pressure, pulse, and others, for a more precise assessment of the impact of hypertension on temperature. While our study focused on specific aspects, the inclusion of these additional measures in future research could offer a more comprehensive view of the physiological changes associated with hypertension. Furthermore, we were constrained by the limited battery life of the telemetry monitoring sensor. This limitation impacted the duration of our observations, and, as a result, we recommend extending monitoring periods in future studies to gain deeper insights into the long-term effects of hypertension on the observed physiological variables. Despite these constraints, we believe that our study contributes valuable insights to the existing literature, and we recognize the importance of addressing these limitations to guide and inform future research. In future studies, we would also recommend detailed measurements of melatonin levels to better understand the mechanisms of its production and regulation, including the influence of different lighting conditions and light spectral composition. Subsequent studies with larger sample sizes, incorporation of additional hemodynamic and other parameters, and extended monitoring periods will likely enhance our understanding of the intricate relationships between hypertension and temperature regulation.

## 5. Conclusions

The daily rhythms of Tcore and locomotor activity persisted in WKY and SHR during periods of standard daily lighting (12–12 h) following frequent changes in experimental lighting regimes. Tcore and activity changes were observed in both groups. A relationship between Tcore and activity was observed with weak correlation. WKY exhibited more pronounced signs of adaptive variation and desynchronization (phase shift) compared to SHR, which might be associated with a broader range of functional capabilities of the organism without cardiovascular pathology.

## Figures and Tables

**Table 1:** Tcore values in 24 h, darkness, and light by periods and groups of rats (WKY and SHR). Data are expressed as means ± SEM.

Periods	Groups	Temperature in 24 h (Mean ± SEM, °C)	Temperature in Darkness (Mean ± SEM, °C)	Temperature in Light (Mean ± SEM, °C)
period_1	WKY	37.397 ± 0.010	38.140 ± 0.007 ⬥	36.655 ± 0.005 ⬥
period_1	SHR	37.380 ± 0.007	37.717 ± 0.011 ⬥	37.044 ± 0.005 ⬥
period_2	WKY	37.398 ± 0.008 ⬥	37.774 ± 0.007 ⬥✱➊	37.022 ± 0.010 ⬥✱➊
period_2	SHR	37.423 ± 0.009 ⬥	38.132 ± 0.007 ⬥✱➊	36.715 ± 0.005 ⬥✱➊
period_3	WKY	37.339 ± 0.009 ⬥✱➊	37.778 ± 0.008 ⬥➊	36.884 ± 0.011 ⬥✱➊
period_3	SHR	37.371 ± 0.010 ⬥✱➊	38.031 ± 0.008 ⬥✱➊	36.711 ± 0.008 ⬥✱➊
period_4	WKY	37.320 ± 0.008 ⬥➊	37.816 ± 0.007 ⬥✱➊	36.824 ± 0.009 ⬥➊
period_4	SHR	37.587 ± 0.008 ⬥✱➊	38.165 ± 0.006 ⬥✱➊	37.010 ± 0.004 ⬥✱➊
period_5	WKY	37.348 ± 0.006 ⬥✱	37.734 ± 0.007 ⬥✱➊	36.963 ± 0.006 ⬥✱➊
period_5	SHR	37.415 ± 0.009 ⬥✱	38.033 ± 0.009 ⬥✱➊	36.798 ± 0.008 ⬥✱➊
period_6	WKY	37.408 ± 0.008 ⬥✱	37.836 ± 0.006 ⬥✱➊	36.981 ± 0.010 ⬥✱➊
period_6	SHR	37.677 ± 0.008 ⬥✱➊	38.255 ± 0.005 ⬥✱➊	37.101 ± 0.004 ⬥✱➊

⬥ *p* < 0.05 WKY vs. SHR in one period, ✱ *p* < 0.05 vs. previous period, ➊ *p* < 0.05 vs. first period.

**Table 2:** Activity values in 24 h, darkness, and light by periods and groups of rats (WKY and SHR). Data out of IQR was filtered out and expressed as means ± SEM.

Periods	Groups	Activity in 24 h (Mean ± SEM, Units)	Activity in Darkness (Mean ± SEM, Units)	Activity in Light (Mean ± SEM, Units)
period_1	WKY	0.638 ± 0.017 ⬥	2.050 ± 0.055 ⬥	0.198 ± 0.009
period_1	SHR	0.586 ± 0.016 ⬥	1.388 ± 0.043 ⬥	0.189 ± 0.009
period_2	WKY	0.614 ± 0.016	1.484 ± 0.046 ⬥✱➊	0.203 ± 0.01
period_2	SHR	0.615 ± 0.016	1.628 ± 0.046 ⬥✱➊	0.183 ± 0.009
period_3	WKY	0.599 ± 0.016 ➊	1.372 ± 0.044 ⬥➊	0.190 ± 0.01 ⬥
period_3	SHR	0.592 ± 0.016	1.853 ± 0.049 ⬥✱➊	0.140 ± 0.008 ⬥✱➊
period_4	WKY	0.540 ± 0.015 ✱➊	1.400 ± 0.044 ⬥➊	0.162 ± 0.008 ➊
period_4	SHR	0.539 ± 0.015 ✱	1.494 ± 0.044 ⬥✱➊	0.154 ± 0.008 ➊
period_5	WKY	0.542 ± 0.015	1.388 ± 0.044 ⬥➊	0.159 ± 0.008 ➊
period_5	SHR	0.578 ± 0.016	1.556 ± 0.045 ⬥➊	0.165 ± 0.008
period_6	WKY	0.705 ± 0.017 ⬥✱➊	1.649 ± 0.048 ⬥✱➊	0.237 ± 0.011 ⬥✱➊
period_6	SHR	0.619 ± 0.016 ⬥✱➊	1.988 ± 0.051 ⬥✱➊	0.155 ± 0.008 ⬥➊

⬥ *p* < 0.05 WKY vs. SHR in one period, ✱ *p* < 0.05 vs. previous period, ➊ *p* < 0.05 vs. first period.

**Table 3:** Correlation of Tcore and activity data in darkness and light by periods and groups. The evaluation periods under standard lighting conditions were subsequent to days exposed to various experimental lighting conditions: period_1 followed a postoperative recovery week, period_2 followed days following a 20 h light and 4 h darkness regime, period_3 followed a monochromatic light regimen, period_4 followed days affected by light pollution, period_5 followed a regimen of bright light therapy, and period_6 followed continuous illumination (24:0 h). Data out of IQR was filtered out.

Day Phase	Periods	Groups	Spearman Test	*p*_Value
Darkness	period_1	SHR	0.02	0.40
Darkness	period_1	WKY	0.13	6.14 × 10^−13^
Darkness	period_2	SHR	0.18	1.80 × 10^−21^
Darkness	period_2	WKY	0.24	2.93 × 10^−42^
Darkness	period_3	SHR	0.23	6.25 × 10^−34^
Darkness	period_3	WKY	0.18	2.32 × 10^−22^
Darkness	period_4	SHR	0.18	6.43 × 10^−21^
Darkness	period_4	WKY	0.21	1.15 × 10^−30^
Darkness	period_5	SHR	0.27	1.91 × 10^−47^
Darkness	period_5	WKY	0.31	1.34 × 10^−62^
Darkness	period_6	SHR	0.26	5.91 × 10^−43^
Darkness	period_6	WKY	0.31	1.25 × 10^−70^
Light	period_1	SHR	-0.02	0.31
Light	period_1	WKY	0.12	3.20 × 10^−12^
Light	period_2	SHR	0.03	0.14
Light	period_2	WKY	0.24	9.08 × 10^−43^
Light	period_3	SHR	0.11	3.60 × 10^−10^
Light	period_3	WKY	0.23	5.94 × 10^−39^
Light	period_4	SHR	0.10	3.92 × 10^−08^
Light	period_4	WKY	0.17	4.33 × 10^−22^
Light	period_5	SHR	0.15	2.80 × 10^−19^
Light	period_5	WKY	0.12	2.69 × 10^−11^
Light	period_6	SHR	0.08	6.43 × 10^−07^
Light	period_6	WKY	0.36	2.80 × 10^−100^

## Data Availability

Data available on request due to technical restrictions. The data presented in this study are available on request from the corresponding author. The data are not publicly available due to legal issues and restricted international access.
